# The Effects of the COVID-19 Pandemic on Mother-Infant Mental Health Relationship

**DOI:** 10.1192/j.eurpsy.2022.59

**Published:** 2022-09-01

**Authors:** T. Kurimay

**Affiliations:** North Centre Buda New Saint John Hospital and Oupatient Clinic, Buda Family Centre Mh Centre Department Of Psychiatry, Teaching Dep. Of Semmelweis University, Budapest, Hungary

## Abstract

Maternal health cannot be separated from infant, child and adolescent health, which includes mental health as well. Expecting mothers go through a number of changes during their pregnancy. Due to the specific alterations of their physique and immune system, pregnant mothers are more vulnerable to the Covid-19 infection. This highlights the importance of the vaccinations in their cases. During the pandemic, mental health problems such as anxiety, depression and stress aroused in greater numbers. This affected mothers, and younger children as well. Expecting mothers, without pre-existing mental disorder (>50%) reported a weightier level of anxiety in their first trimester. Also, infants can suffer developmental disadvantages, as their infected mothers are separated from them. Even though evidence is not yet clear in this topic, vertical transmission seems to be fairly uncommon. Treatment guidelines, that could help Covid-19 infected mothers to handle their infants, are scarce. Hence the importance of telehealth has started to be outlined. Separation from the children might be necessary, while the mental health of mother and infant is continuously screened, since the long-term consequences of the symptoms are still unknown. Hence, prevention is imperative to avoid any negative effects. Even still, WHO advises mothers to breastfeed safely, with good respiratory hygiene, emphasizing the importance of skin-to-skin contact of newborns and sharing the room with them. On policy level: investment into pre-, peri-, post-natal care, family supporting national programs, inter-sectoral collaborations, monitoring and research are important elements of prevention and treatment efforts during the Epidemic and the post-Covid-19 era.

**Disclosure:**

No significant relationships.

